# Evaluation of the effect of a midwife-led online program using cognitive behavioral therapy for pregnant women at risk for anxiety disorder in Japan: A pilot randomized controlled trial

**DOI:** 10.1371/journal.pone.0281632

**Published:** 2023-05-10

**Authors:** Aiko Okatsu, Ayako Kanie, Yaeko Kataoka

**Affiliations:** 1 Midwifery, School of Nursing, St. Luke’s International University, Akashi-cho, Tokyo, Japan; 2 Center for Cognitive Behavioral Therapy, National Center of Neurology and Psychiatry, Kodaira-Shi, Tokyo, Japan; 3 Psychiatry, The University of Tokyo, Bunkyo-ku, Tokyo, Japan; Brown University, UNITED STATES

## Abstract

**Aim:**

This study aimed to conduct a pilot randomized controlled trial (RCT) to examine the appropriateness and feasibility of a midwife-led cognitive behavioral therapy (CBT)-based, three-session program delivered remotely to pregnant women at risk for anxiety disorders.

**Methods:**

The study design was a pilot RCT. Outcome was the difference between the two groups in the change in generalized anxiety disorder-7 (GAD-7), Kessler6 (K6) and Edinberg Postnatal Depression Scale (EPDS) etc. Recruitment was conducted from August 2020 to July 2021 in clinics and web-based survey monitors in Japan, with follow-up through November 2021.

**Results:**

This program involving 63 pregnant women were administered. Although the intervention was remote, a total of three sessions was completed during pregnancy. The change in GAD-7 scores from pre- to 1 month postpartum, was mean -1.77 in the intervention group and mean -0.97 in the control group, with a p-value of .521, indicating no significant difference between the two groups, although GAD-7 scores were lower in the intervention group. The change in K6 score from pre- to 1 month postpartum, was mean -3.55 in the intervention group and mean -1.62 in the control group, with a p-value of .168, indicating no significant difference between the two groups, although the intervention group showed a greater decrease. In particular, in primiparas, the change in GAD-7 scores in the intervention group was large, and some expressed a desire for a postpartum session, suggesting that a follow-up session after delivery may be effective. In multiparas, the control group showed an increase in both GAD-7 and K6 scores from late pregnancy to 1 month postpartum, while the intervention group showed a decrease in scores.

**Conclusion:**

The program was implemented using CBT conducted by midwife, anxiety decreased in primiparas. In future RCTs, it was suggested that additional postpartum sessions may be effective.

**Trial registration:**

UMIN Clinical trial registry ID: UMIN000040304.

## Introduction

The incidence of postpartum depression has been reported as 10%–15% in the U.S. [1]. A systematic review focused on the prevalence of perinatal depression reported the global average for the incidence of perinatal depression as 17.7%, with that in Japan being 10.3%–27.3% [2]. This is influenced by changes in endocrine balance, family roles, and role changes in society, which may have been present in the past but have recently received more attention. Furthermore, the prevalence of perinatal depression has been reported to be higher due to the COVID-19 epidemic [3]. Maternal depression up to 1 year postpartum is associated with poor behavioral, cognitive, and emotional outcomes for children [4, 5]. This suggests that interventions that effectively reduce maternal depressive symptoms may also improve child outcomes [6]. Therefore, improving support for maternal mental health is an urgent issue.

Recently, it has been shown that 39% of patients with GAD-7 met the criteria for depression [7]. This means that in the perinatal field, it is important to intervene in anxiety disorders to prevent depression. The UK National Institute for Health and Care Excellence published prenatal and postnatal mental health guidelines [8], that recommend self-help based on CBT for mild to moderate depression during pregnancy and postpartum, as well as for mild anxiety disorders. Interventions using CBT in pregnancy may be delivered by psychiatrists, clinical psychologists, junior mental health workers trained in CBT for 1 year, and other trained professionals [9, 10]. However, expectant mothers themselves must be willing to receive psychotherapy. Pregnant women can be difficult to access because of minor problems and other changes in their physical condition. In addition, the prolonged COVID-19 epidemic has contributed to the isolation of pregnant women and mothers, making it increasingly difficult for them to visit a health care provider due to the problem of infection. The experience of health worry and sadness associated with COVID-19 has been found to potentially increase the occurrence of symptoms of mental health problems [11]. If midwives trained in delivering CBT can demonstrate the feasibility and effectiveness of CBT-based interventions for pregnant women, it may contribute to improving perinatal mental health.

In this study, we aimed to develop a midwife-led intervention program using CBT for pregnant women at risk for anxiety disorders to examine the preliminary effects and feasibility of the program and inform the sample size calculation in a pilot randomized controlled trial.

## Materials and methods

### Study design

This study was a pilot RCT. Sixty pregnant women at risk for anxiety disorders were randomly assigned to an intervention group that received the CBT intervention and a control group that received usual care.

### Participants

Participants were pregnant women who met the study eligibility criteria: (1) aged 20 years or older; (2) after 22 weeks of pregnancy; (3) scored between 5 and 14 points on the GAD-7 generalized anxiety disorder assessment tool; and the ability to speak, read and write in Japanese. Participants were excluded if they (1) were undergoing hospitalization for psychiatric disorders or (2) were diagnosed with complications of pregnancy and required medical treatment in the hospital. After the beginning of the program, participants were also excluded if they: (1) developed pregnancy complications that required hospitalization; (2) delivered without completion of the interventions; (3) had increased symptoms of anxiety or depression observed during the intervention and a psychiatrist was consulted regarding the discontinuation of the intervention.

### Program overview

The program was based on the theory of CBT, which encompasses the idea of reducing stress in one’s life, work, and relationships by understanding one’s habits of thinking and behavior and adjusting one’s cognitive and behavioral patterns [12]. The program comprised an overview of CBT, an image of postpartum life, and CBT work that assumed postpartum life. For example, the work covered situations that simulated the postpartum situation that were conducted during pregnancy to simulate postpartum life. The aim was to allow participants to practice cognitive restructuring and behavioral change trials while understanding CBT in situations of emotional turmoil that occur in private life, and acquire skills through repeated CBT work while reflecting on their behavior in the sessions.

The interventionist had studied CBT for approximately two years at an institution specializing in CBT. The intervention was conducted in a series of simulated sessions and supervised by an expert. During the course of the intervention period, the quality of the intervention was ensured through simulated sessions and supervision.

The program comprised three individual 30-minute sessions during the pregnancy period. The sessions covered psychoeducation and CBT, using pamphlets and in Japanese. Follow-up emails were sent 2 weeks after completion of the program and at 1, 2, and 3 weeks after delivery. The sessions were conducted remotely because of COVID-19 measures. The session topics are detailed below.

Session 1: The Structure of Emotions

This session explained mental health in the perinatal period, the structure of emotions, and how to solve problems using CBT.

Session 2: Baby’s Crying and Breastfeeding

This session focused on a four-frame cartoon related to a baby crying and breastfeeding, which was discussed using CBT.

Session 3: Relationships during childcare

The final session included four-panel cartoons related to husbands, coworkers, and older children that were discussed using CBT.

Follow-up emails

The purpose of these emails was to remind participants to incorporate the skills learned during the CBT program into their daily lives. No reply was required.

### Study procedure

#### Recruitment and randomization

Recruitment occurred at obstetric clinics and via a web survey monitor from August 2020 to July 2021, and follow-up was conducted until November 2021. The clinics were recruited at five locations in Tokyo or its suburbs, and the web-based survey monitors were recruited in Japan. The study content was presented to potential participants in writing, and those who were interested in the study were asked to complete the GAD-7. Participants who met the eligibility criteria and did not meet any of the exclusion criteria received another explanation about the study in writing and their consent was obtained. At this point, study IDs were assigned and a corresponding table was prepared. Random allocation software was used to perform stratified block random assignment of primipara and multipara participants with a block size of 4. Because of the computer-based software allocation, concealment was ensured. The study was not blinded to participants, interveners, or evaluators.

#### Intervention group

Pregnant women were informed by the research assistant staff that they were in the intervention group and were sent a pre-program questionnaire by mail. After being collected by return mail, CBT pamphlet were mailed to them. At a date and time arranged by email, the interventionist conducted a CBT session remotely with each participant using the pamphlet and through video call function software. The date and time for the next session was determined at that time or by subsequent email. After the third session, a post-program evaluation questionnaire was mailed to participants and later collected by return mail. A follow-up email was sent 2 weeks after the third session. In this email, the researcher asked the participants to contact her if they were going to give birth; if they did not, the researcher determined the expected delivery date and sent an email. Further follow-up emails were sent at 1 week, 2 weeks, and 3 weeks after the day of delivery. A postpartum questionnaire was mailed out around 1 month after delivery, and completed questionnaires were collected. In addition, during the sessions, interveners worked from private rooms in the laboratory or at home, and data collection was managed in a secure room at the university. In addition, the researcher explained to the intervention group that since the study was in progress, they were prohibited from talking about the program or sharing the contents of the pamphlet.

Session interventions were performed by a midwife at the advanced level.

#### Control group

Pregnant women were informed that they were the control group and sent a pre-program questionnaire that was later collected. A second questionnaires was then mailed out at around 33–36 weeks of pregnancy to be completed and returned, which was around the time when the intervention group would have finished their CBT sessions. The researcher asked the control group participants to contact her when they were going to give birth; if they did not, the researcher determined the expected delivery date and sent an email. At around 1 month postpartum, a postpartum questionnaire was mailed to participants and later collected. Control group participants then received the CBT pamphlet used for the intervention group via mail.

#### Interventionists

The principal investigator, who is a midwife, mainly conducted the intervention. All other interveners were certified as advanced midwives. A system was put in place whereby a research collaborator with advanced midwifery certification could intervene if the principal investigator was unable to do so. The principal investigator and research collaborators had received training and supervision from CBT experts for more than 1 year.

#### Sample size

The recommended sample size for a pilot study is at least 50 participants [13]. In this study, a total of 60 pregnant women (in both groups) that had GAD-7 scores of 5–14 points were included to account for dropout.

### Outcomes

#### Primary outcome

The primary outcome was the change in participants’ anxiety level (GAD-7 score). The GAD-7 was developed as a brief assessment tool for generalized anxiety disorder, which is recommended by the NICE guidelines as an assessment scale for depression and is a 7-item scale on a four-point scale (0–21 points) [14]. The Japanese version has been validated for validity and effectiveness and is considered valid [15].

#### Secondary outcomes

There were four secondary outcomes, as follows.

1. Changes in the degree of depression and anxiety (K6) [16].
  The K6 is a psychological scale developed to screen for mental health in the general adult population. It is a 6-item tool with responses on a five-point scale (total 0–24 points) measuring depression and anxiety. It has been confirmed to be sufficiently reliable and valid [17, 18].
2. Degree of risk for postpartum depression (EPDS) at 1 month postpartum.
  The EPDS was developed by Cox et al. in the UK [19] and translated by Okano et al. [20]. The scale has 10 items with response on a four-point scale, and the total score (0–30 points) is used to assess the risk for postpartum depression, using a cutoff of 9 points; a score of 9 points or higher is considered high risk for postpartum depression. In addition, confirmatory and exploratory factor analyses revealed a three-factor structure comprising depression, anxiety, and loss of pleasure factors [21].
3. Change in self-efficacy regarding CBT response.
  This program was based on CBT theory, whereby in the process of trying the skills repeatedly, the skills become one’s own and can be used in daily life. Therefore, we measured participants’ efficacy in accepting and using these skills in daily life. As no existing scales were available, we developed original self-efficacy assessment tool for CBT response in the perinatal period. The construct comprised three sub-concepts (21 items in total): value perception of CBT (seven items), self-efficacy for cognitive restructuring (seven items), and self-efficacy for behavioral change (seven items), on a five-point scale (21–105).
  To evaluate the construct validity of the scale, six CBT experts, including a psychiatrist and a psychologist, were consulted. To establish the content validity, we asked 10 participants (five midwives, two nurses, one childminder, and two office workers) to classify the items and confirm to which of the three subscales each question belonged.
4. Program satisfaction (Japanese version of the Client Satisfaction Questionnaire-8; CSQ-8J).
  The CSQ-8J has eight items on a four-point scale (8–32), with higher scores indicating greater satisfaction. The reliability and validity of the CSQ-8J have been confirmed [22].

#### Participants, characteristics

We collected basic information about participants, including age, gestational weeks, childbirth history, history of previous pregnancies and pregnancy complications, background to the pregnancy, family structure, education level, work status, household income, and mode of delivery.

### Data analysis

Statistics were performed using IBM SPSS statistics ver.27. For comparison of the change in each outcome, the mean of the change was calculated, the normality of each was checked, and if normally distributed, a t-test was performed. The effect size (Cohen’s d) was calculated from the mean and standard deviation values. Alternatively, a Mann-Whitney U test was performed in the case of a non-normal distribution. Subgroup analysis by parity (primipara and multipara) was also used to compare changes in each outcome, the mean of the change was calculated, the normality of each was checked, and if normally distributed, a t-test was performed. The effect size (Cohen’s d) was calculated from the mean and standard deviation values. Participants’ satisfaction with the program was assessed for the mean CSQ-8J score using a t-test.

### Ethical considerations

This study was conducted in accordance with the Declaration of Helsinki and the Ethical Guidelines for Medical and Health Research involving Human Subjects [23]. This study was approved by the Research Ethics Review Committee of University A (20-A012). The researcher explained the study purpose and procedure to participants including the freedom to withdraw from the study and obtained their written consent to participate.

## Results

### Flow of participants

**Fig 1:**
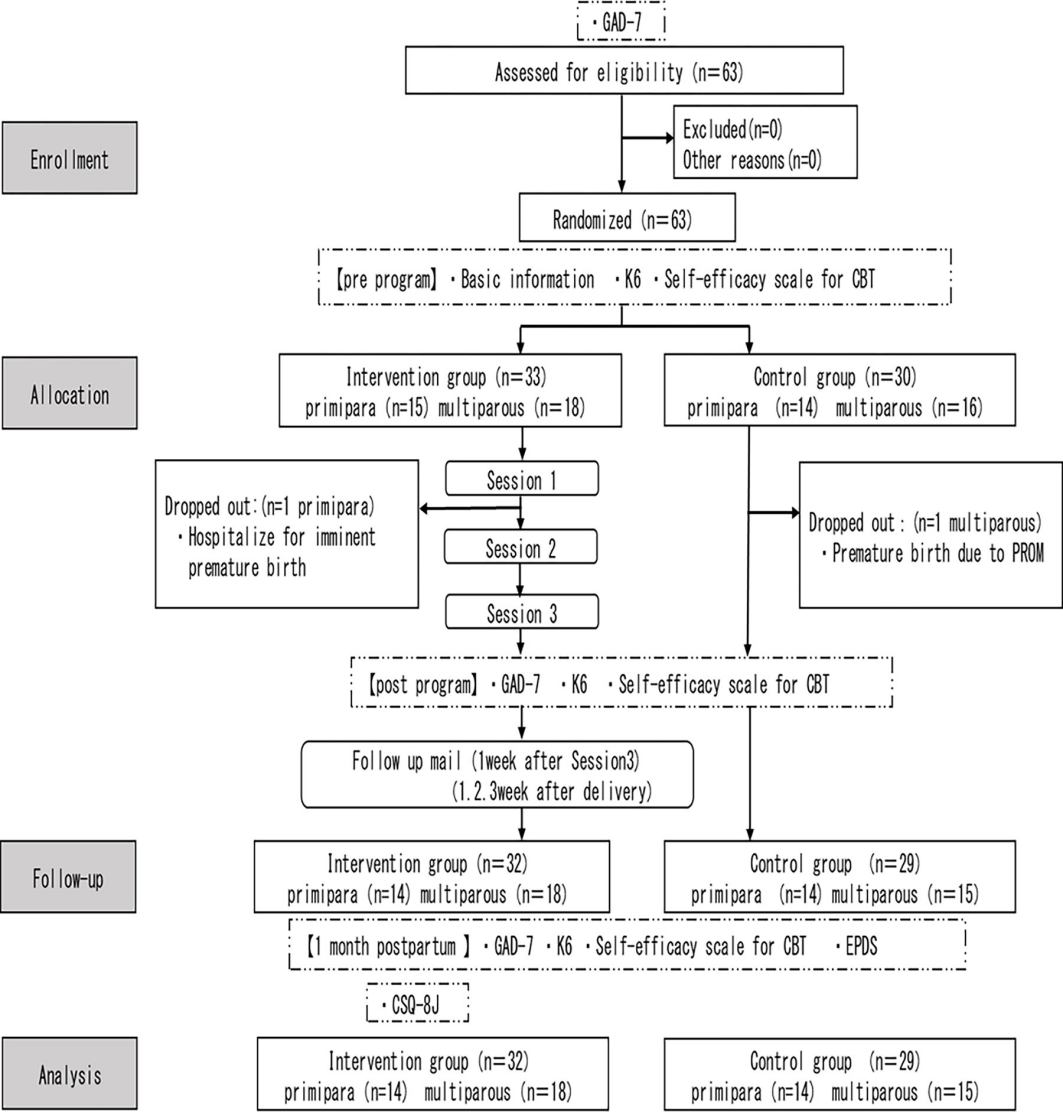
The flow of participants.

The flow of participants is shown (see Fig 1). In total, 63 participants who met the inclusion criteria gave consent to participate in this study. Thirty-three participants were assigned to the intervention group, but one participant dropped out because of hospitalization for impending preterm labor; the remaining 32 participants responded to the questionnaire at 1 month postpartum (97% follow-up rate). Thirty participants were assigned to the control group. One participant dropped out because of preterm labor during the study; the remaining 29 participants responded to the questionnaire at 1 month postpartum (follow-up rate: 96.7%). Finally, 32 intervention group participants and 29 control group participants were included in the analyses. Among the 32 participants in the intervention group, 14 were primipara and 18 were multiparous. Among the 29 women in the control group, 14 were primipara and 15 were multiparous.

There was no apparent worsening of psychiatric symptoms or interruption of the intervention during the pregnancy period because of harm to the course of the pregnancy.

### Baseline characteristics

**Fig 2:**
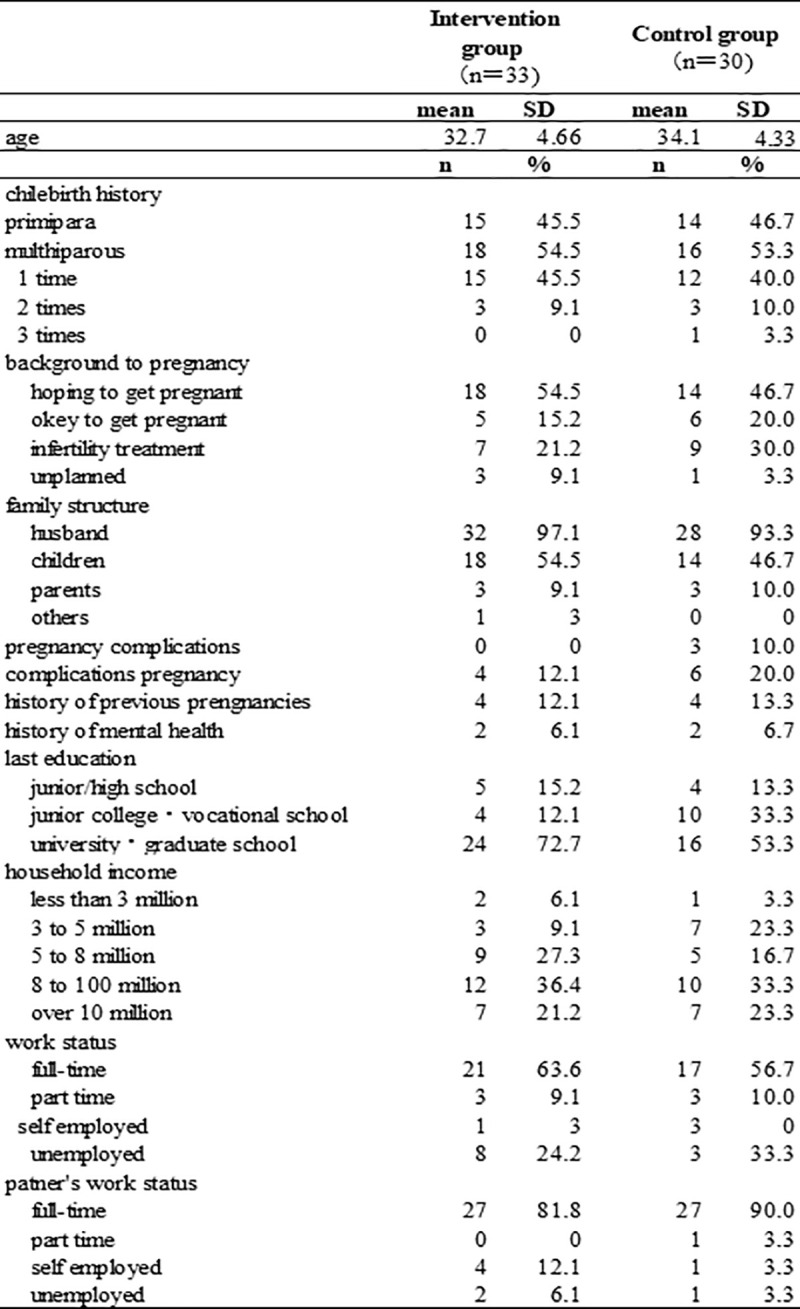
Characteristics of participants.

**Fig 3:**
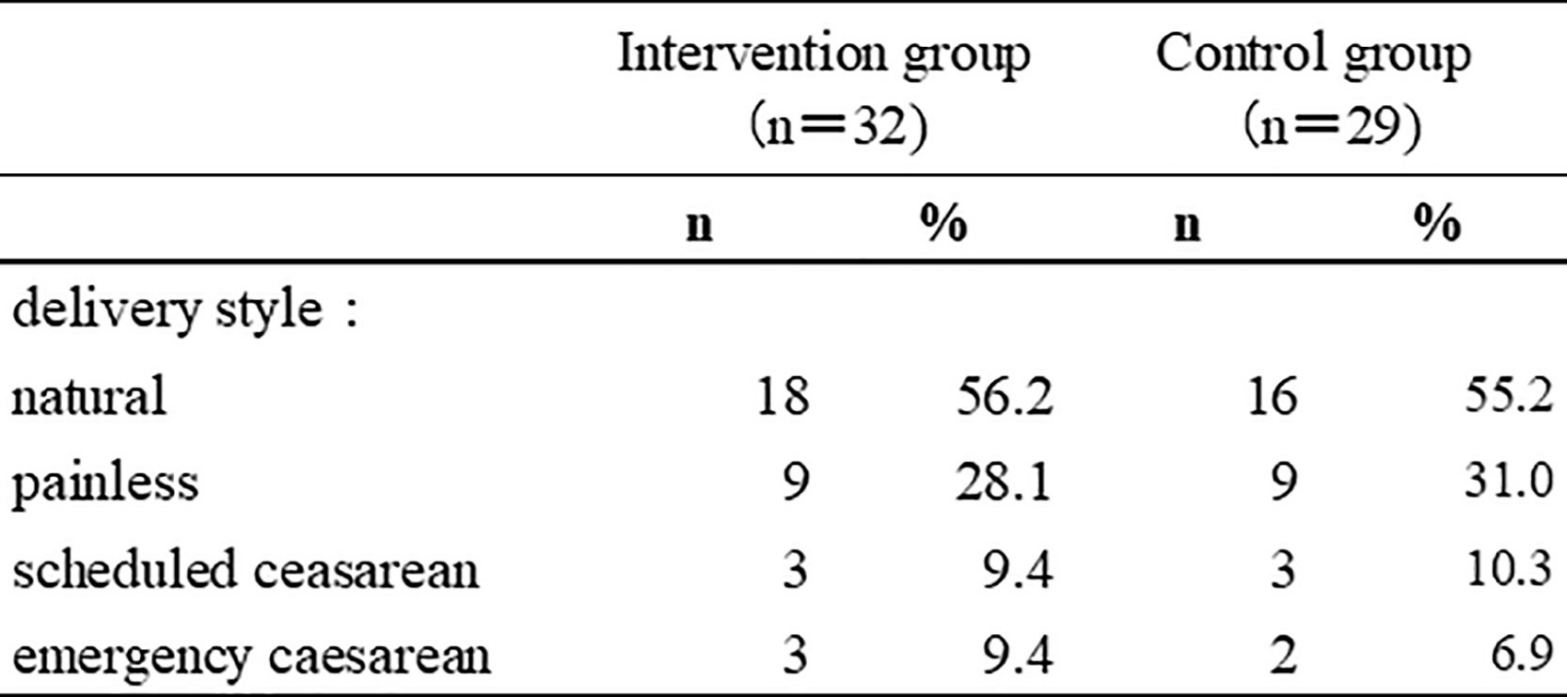
The final style of delivery.

The characteristics of each group before the intervention are shown in the table. (see Fig 2) The mean age was 32.1 years (standard deviation [SD] = 3.11 years) in the intervention group and 34.1 years (SD = 4.33 years) in the control group. After infertility treatment, the rate was 20%–30%. The reported pregnancy complications did not significantly differ between the two groups and had no effect on dropout. Most participating pregnant women had nuclear families and had graduated from college or technical school, and few had a low household income. More than half of the participants were working full-time, and 53.1% were employed before and after the birth of their first child, which was similar to the general situation in Japan. There were no other obvious differences in participants’ characteristics between the two groups. The mean gestational age at recruitment was 29 weeks in both groups. Participants’ final style of delivery is shown (see Fig 3).

### Outcomes

#### Primary outcome

**Fig 4:**
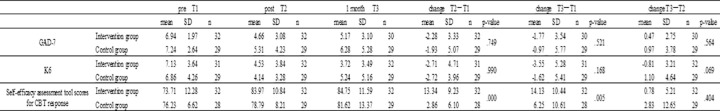
Changes in GAD-7, K6, Self-efficacy assessment tool scores for CBT response.

The primary outcome was change in the level of anxiety (GAD-7 score). Since a normal distribution was confirmed, a t-test was performed. The changes in GAD-7 scores and t-test results are shown in Fig 4. The mean change (T3–T1) was mean−1.77 (SD = 3.54) in the intervention group and mean−0.97 (SD = 5.76) in the control group. Although the change was greater in the intervention group than in the control group, the difference was not significant (p = .521, effect size: d = .159). The mean amount of change (T2–T1) was mean−2.28 (SD = 3.33) in the intervention group and mean−1.97 (SD = 5.07) in the control group, but there was no significant difference between the two groups (p = .749, d = .078).

The level of anxiety is likely to vary depending on the stage of pregnancy and postpartum period. Therefore, we conducted a two-way analysis of variance (ANOVA) between the timing and the presence of the intervention. We found no interaction between timing and the presence of the intervention. The main effect was confirmed only for the time period, and the results of multiple comparisons (Bonferroni) showed that pre- (T1) > post- (T2) at α = 0.05. The results of the analysis for the intervention and control groups showed that the intervention group differed significantly in the degree of anxiety depending on the time of year (F(2, 58) = 7.838, p = .001), whereas the target group did not differ significantly in the degree of anxiety depending on the time of year (F(1.663, 46.553) = 2.214, p = .129).

### Secondary outcomes

**Fig 5:**
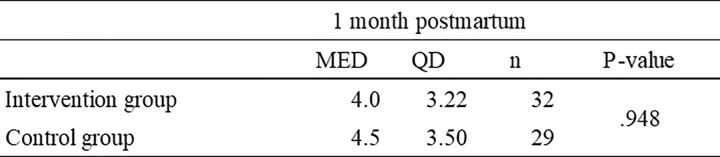
Level of risk of postpartum depression (EPDS) at 1 month postpartum.

**Fig 6:**
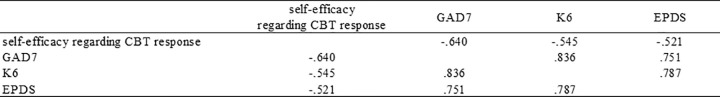
Correlation between self-efficacy regarding CBT response and GAD-7, K6, EPDS.

1. Changes in the level of depression and anxiety (K6 score).
  Since a normal distribution was confirmed for K6, a t-test was performed. The change in the K6 scores and results of the t-tests are shown in Fig 5. The mean change (T3–T1) was mean−3.55 (SD = 5.28) in the intervention group and mean−1.62 (SD = 5.41) in the control group. There was no significant difference between the two groups (p = .168), but the intervention group was more reduced than the control group. The effect size was d = .361. The mean change (T2–T1) was mean−2.71 (SD = 4.71) in the intervention group and mean−2.72 (SD = 3.96) in the control group. There was no significant difference between the two groups (p = .168). The effect size was d = .361.
  For the K6 scores, the results of two-way ANOVA between the timing and the intervention showed no interaction between the timing and the intervention. The main effect was confirmed only for timing, and the results of multiple comparisons (Bonferroni) showed that, atα = 0.01, pre- (T1) > post- (T2) and pre- (T1) > 1 month postpartum (T3). The results of the analysis in the intervention and control groups respectively showed that the degree of depression and anxiety in both the intervention and control groups differed significantly depending on the time of year (F(1.599, 47.977) = 10.521, p≺.001; F(2, 56) = 4.914, p = .011).
2. Level of risk for postpartum depression (EPDS score) at 1 month postpartum.
  The mean EPDS scores were calculated at 1 month postpartum. Since the EPDS was non-normally distributed, a Mann-Whitney-U test was performed. The median was 4.5 for the intervention group and 4 for the control group, there was no significant difference between the two groups (p = .948) (see Fig 5).
3. Change in self-efficacy for CBT response (self-efficacy assessment tool for CBT response).
  As the self-efficacy assessment tool for CBT response in the perinatal period was developed independently, we confirmed the internal consistency. The Cronbach’s α reliability coefficients for the 21-item scale were .874 before the intervention, .905 after the intervention, and .933 at 1 month postpartum. Spearman’s rank correlation coefficient was used to check whether the rating tables correlated with other scales (see Fig 6). The correlation coefficients were −.640 with the GAD-7, −.545 with the K6, and −.521 with the EPDS, which confirmed a moderate correlation.

Changes in the level of self-efficacy assessment tool scores are shown in Fig 4. Since a normal distribution was confirmed, a t-test was performed. The pre- and post-program (T2–T1) change was mean13.34 (SD = 9.23) in the intervention group and mean2.86(SD = 6.10) in the control group; the change in the intervention group was significantly higher than that in the control group (p < .001, d = 1.289). The change from pre-program to 1 month postpartum was mean14.13 (SD = 10.44) in the intervention group and mean6.25 (SD = 10.61) in the control group, with the change in the intervention group being significantly higher than in the control group (p = .005, d = .749). The amount of change from post-program to 1 month postpartum was almost unchanged in the intervention group.

#### Subgroup analysis

For each outcome, subgroup analysis was performed based on whether participants were primipara or multipara.

**Fig 7:**
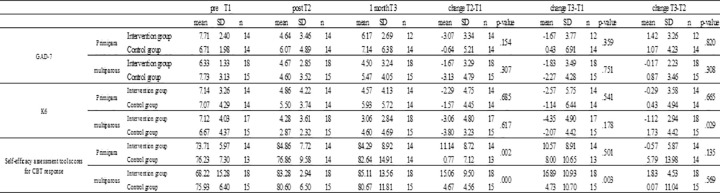
Changes in GAD-7, K6, Self-efficacy assessment tool scores for CBT response by primipara and multiparous.

1. Change in the level of anxiety (GAD-7 score).
  The results of GAD-7 scores are shown in Fig 7. Since a normal distribution was confirmed, a t-test was performed. In primiparas, the amount of change (T2–T1) pre- and post-program was mean−3.07 (SD = 3.34) in the intervention group and mean−0.64 (SD = 5.21) in the control group, with no significant difference between the two groups (p = .154). However, the amount of change was greater in the intervention group and the effect size was moderate (d = .53). The change from pre-program to 1 month postpartum (T3–T1) was mean−1.67 (SD = 3.77) in the intervention group and mean0.43 (SD = 6.91) in the control group, which was not significant (p = .359). However, the change was greater in the intervention group than in the control group (d = .35). The amount of change (T3–T2) from post-program to 1 month postpartum was 1.42 (SD = 3.26) in the intervention group and mean1.07 (SD = 4.23) in the control group. The intervention group had more anxiety than the control group, but there was no significant difference between the two groups (p = .820).
  In multiparas, the amount of change between pre- and post-program (T2–T1) was mean−1.67 (SD = 3.29) in the intervention group and mean−3.13 (SD = 4.79) in the control group, with no significant difference between the two groups (p = .307). However, the change was greater in the control group than in the intervention group. The amount of change (T3–T1) from pre-intervention to 1 month postpartum was mean−1.83 (SD = 3.49) in the intervention group and mean−2.27 (SD = 4.28) in the control group. The amount of change was greater in the control group than in the intervention group, but there was no significant difference between the two groups (p = .751). The amount of change from pre-program to 1 month after delivery was mean−0.17 (SD = 2.23) in the intervention group and mean0.87 (SD = 3.46) in the control group. This indicated that the control group had more anxiety than the intervention group, but the difference was not significant (p = .308). The amount of change in menstruating women was greater in the control group, but the effect size could not be calculated.
2. Changes in the level of depression and anxiety (K6 score).
  The results of K6 scores are shown in Fig 7. Since a normal distribution was confirmed, a t-test was performed. In primiparas, the amount of change (T2–T1) pre- and post-program was mean−2.29 (SD = 4.75) in the intervention group and mean−1.57 (SD = 4.45) in the control group. The amount of change was greater in the intervention group than in the control group (d = .156). The change from pre-program to 1 month postpartum was mean−2.57 (SD = 5.75) in the intervention group and mean−1.14 (SD = 6.44) in the control group, with the intervention group showing a larger change than the control group (d = .233).
  In multiparas, the change between pre- and post-program (T2–T1) was mean−3.06 (SD = 4.80) in the intervention group and mean−3.80 (SD = 3.23) in the control group, but there was no significant difference between the two groups (p = .617). The change from pre-intervention to 1 month postpartum (T3–T1) was mean−4.35 (SD = 4.90) in the intervention group and mean−2.07 (SD = 4.42) in the control group, with the intervention group showing a greater change than the control group (d = .509). The amount of change from post-intervention to 1 month postpartum was mean−1.12 (SD = 2.94) in the intervention group and mean1.73 (SD = 4.42) in the control group. The control group had more anxiety than the intervention group, but the change in the intervention group was significant (p = .029).
3. Changes in the level of self-efficacy for CBT response (Self-efficacy assessment tool scores for CBT response)
  The results of the self-efficacy assessment tool scores for CBT response are shown in Fig 7. Since a normal distribution was confirmed, a t-test was performed. In primiparas, the amount of change before and after the program was mean11.14 in the intervention group (SD = 8.72) and mean77.00 (SD = 7.12) in the control group. The amount of change was greater in the intervention group than in the control group, and the intervention group showed a significant increase (p = .002). The amount of change from post-program to 1 month postpartum was mean−0.57 (SD = 5.87) in the intervention group and mean5.79 (SD = 13.98) in the control group. There was no significant difference between the two groups (p = .135), but the intervention group had maintained their skills.

In multiparas, the amount of change between before and after the intervention was mean15.06 (SD = 9.50) in the intervention group and mean4.67 (SD = 4.56) in the control group. The amount of change was greater in the intervention group than in the control group, with the intervention group showing a significant increase (p < .000). The change from pre-intervention to 1 month postpartum was 16.89 (SD = 10.93) in the intervention group and mean4.73 (SD = 10.70) in the control group. The intervention group showed a greater change than the control group, and also showed a significant increase (p = .003). The amount of change from the intervention group to 1 month after delivery was 1.83 (SD = 4.53) in the intervention group and mean0.07 (SD = 11.04) in the control group. The intervention group showed a greater increase than the control group, but the difference between the groups was not significant (p = .581).

## Discussion

### Intervention effects

The prevalence of postpartum depression was previously reported as 13.0%–19.2% at 12 weeks postpartum [24]. In addition, it has also been reported that postpartum depression and anxiety are augmented by a decrease in household income and lack of human interaction due to COVID-19 pandemic measures [11]. Therefore, GAD scores in the 1 month postpartum showed an increase in both groups, which can be considered a natural process. However, the fact that at 1 month postpartum, the change in GAD7 scores was less in the intervention group, and that K6 scores did not increase in the intervention group, CBT program may have had a positive impact on postpartum life.

In terms of the self-efficacy of CBT responses, the change in scores between pre- and post-program was significantly higher in the intervention group than in the control group. This suggested that participants were trying to apply the program content to their daily lives after the program. The amount of change from the post-program to 1 month postpartum remained almost the same in the intervention group, and the amount of change from pre-program to 1 month postpartum was significantly higher in the intervention group than in the control group. Although self-efficacy could be further increased by continuing to use the program content, follow-up after childbirth appeared to be necessary because self-efficacy was only maintained. In this study, there were no significant differences in the amount of change in anxiety and depressive symptoms, but the results suggested that the CBT intervention influenced anxiety reduction if it was made a habit.

In primiparas, the GAD-7 scores decreased significantly after the intervention, and small to moderate effect sizes were observed (T2–T1: d = .53 and T3–T1: d = .359). Anxiety disorders were observed among 15.8% of participants during pregnancy and in 17.1% postpartum, of which GAD (which tends to be higher postpartum) accounted for 2.6% during pregnancy and 3.2% postpartum [25]. Although the increase in scores after childbirth can be considered natural, the fact that self-efficacy was maintained from post-program to 1 month after childbirth suggested that sessions after the start of childcare can be used in a timely manner for childcare, and postpartum follow-up sessions may be needed, especially for first-time mothers.

In multiparas, the intervention group continued to show a downward trend in in GAD-7 scores pre- and post-program and at 1 month postpartum, whereas the control group showed an increase in scores 1 month postpartum, similar to primipara participants. There was a significant difference in the change in K6 scores from post-program to 1 month postpartum, with the control group’s scores increasing and the intervention group’s scores decreasing more than post-program. It is thought that the CBT intervention was meaningful for multipara women during pregnancy, as participants received the intervention while taking care of their older children, and the intervention content overlapped with their daily lives, allowing them to understand and immediately practice the skills learned. These results indicated that the intervention tended to differ for primipara and multipara women.

CBT has no side effects and can be used by all pregnant and nursing mothers, meaning the need for CBT is high. Primiparas showed a greater decrease in anxiety scores than multiparas. A meta-analysis of the prevalence of perinatal depression in Japanese women showed the prevalence of postpartum depression 1 month after delivery was 14.3%, with the prevalence of postpartum depression in primiparas reported to be significantly higher than in multiparas [26]. This suggested that by adding a follow-up session after childbirth and increasing the sample size, the CBT intervention may be particularly effective for primiparas.

This study was a pilot RCT, and its primary significance is to provide suggestions for future RCTs. Due to the small sample size did not allow us to test the effectiveness of the intervention. However, it was clear that an effect size was secured and that primiparous was particularly effective, suggesting that the effect will be tested in future RCTs.

### Appropriateness of the intervention

There was only one dropout each in the intervention and control groups, and there was no voluntary discontinuation. In addition, there were no cases in which anxiety was significantly enhanced by the program, and both anxiety and depression scores were lower in the intervention group in terms of outcome. It may be possible for trained midwives to provide a CBT program to pregnant women who are at risk for anxiety disorders but that do not meet the threshold for formal diagnosis.

CBT is generally given weekly or biweekly as a treatment. The mean number of gestational weeks at recruitment in this study was 29 weeks, with few dropouts. As the sessions were completed at around 35 weeks, approximately 30 weeks seems an appropriate time to initiate CBT.

This study was conducted remotely because of measures implemented to mitigate the COVID-19 pandemic. In a situation where face masks were required to be worn, conducting remote sessions made it possible to remove face masks, which offered the advantage of being able to see facial expressions. Remote sessions for CBT delivery may be valuable going forward and will allow confirmation of facial expressions and conditions, while also permitting easy access and infection control. Digital therapy is evolving, and i-CBT has been reported to be effective [27–29], with both face-to-face and remote delivery being possible means of implementation. There is expected to be no difference in effectiveness between face-to-face and remote interventions [30], and given the low dropout rates, remote interventions are considered a promising method.

No participants withdrew from this study and participants’ satisfaction was high. Professional advice on problems encountered during the course of pregnancy and other issues can reduce anxiety, and it is highly significant that midwives, who are in regular contact with women from early pregnancy to postpartum, continue to provide care.

In addition, there was no evidence of deterioration in mental status, which showed that the intervention was not harmful. CBT has an infinitesimally low potential to cause harm, and its validation is highly significant.

### Implications for RCTs

This study was a pilot study design and a moderate effect size was secured. Based on these results, it can be said that there is significance in verifying the results in an RCT. In addition, it may be possible to conduct the sessions in the postpartum period as well, in which case, more effectiveness could be expected.

For the participants, an exploratory analysis was conducted with stratified randomization for primipara and multipara participants. Primiparas showed particularly large fluctuations in anxiety. In a study of Japanese women using the EPDS, primiparas were at high risk for postpartum depression [31], and the factor structure of the EPDS predicted this group had a high risk for anxiety. The amount of change also differed between primiparas and multiparas. Since the degree of risk differs between primiparas and multiparas, and the circumstances of pregnancy and the postpartum period also differ, the intervention effect can be evaluated if separate analyses are conducted for primipara and multipara participants. Therefore, analyses should be conducted separately for primiparas and multiparas. For outcome items, the CBT Response Rating Chart showed negative correlations with the GAD-7 and K6, confirming its internal validity. It can be used with the GAD-7 and K6 in assessing whether it was effective in short-term behavior change.

### Limitations of the study

This study was conducted on Japanese women. It was not possible to examine cases in which race and culture differed. The interventions in this study were conducted by midwives who received training in CBT for about 2 years, but the impact of intervention skills is likely to be significant, and they need to become more skilled with supervision.

### Possibility of generalization

Perinatal mental health problems are caused by pregnancy and lactation, which makes it difficult to use drugs, including sleeping pills. In addition, access to psychosomatic medicine and counseling is difficult during childcare, making it easy for women to become isolated. This study suggests that intervention by midwives may also be effective. CBT is a psychological therapy for which there is ample evidence of effectiveness, and by verifying the effectiveness of CBT programs by midwives in randomized controlled trials, it will be possible for midwives (who are most familiar with pregnant and nursing mothers) to intervene.

## Conclusion

This pilot RCT examined changes in the degree of anxiety and depression in pregnant women at risk for anxiety disorders following implementation of an intervention program using CBT. Implementation of the intervention by midwives reduced changes in anxiety and depression/anxiety in the intervention group. Self-efficacy for CBT responses increased significantly in the intervention group before and after the intervention and persisted until 1 month after delivery. In addition, a different trend was observed among first-time mothers, suggesting that the intervention may be particularly effective for these mothers. In further RCTs, the sample size could be set based on the data obtained in this study, and the intervention program should include additional sessions in the postpartum period.

## Supporting information

S1 Fig (DOCX)

S2 Fig (DOCX)

S3 Fig (DOCX)

S4 Fig (DOCX)

S5 Fig (DOCX)

S6 Fig (DOCX)

S7 Fig (DOCX)

S8 Fig (DOCX)

S9 Fig (DOCX)
