# Supplementary figures and images for: Evaluation of the effect of a midwife-led online program using cognitive behavioral therapy for pregnant women at risk for anxiety disorder in Japan: A pilot randomized controlled trial
Source: PLoS One. 2023 May 10;18(5):e0281632. doi: 10.1371/journal.pone.0281632 (PMC10171600; doi:10.1371/journal.pone.0281632)

Figue.2

*
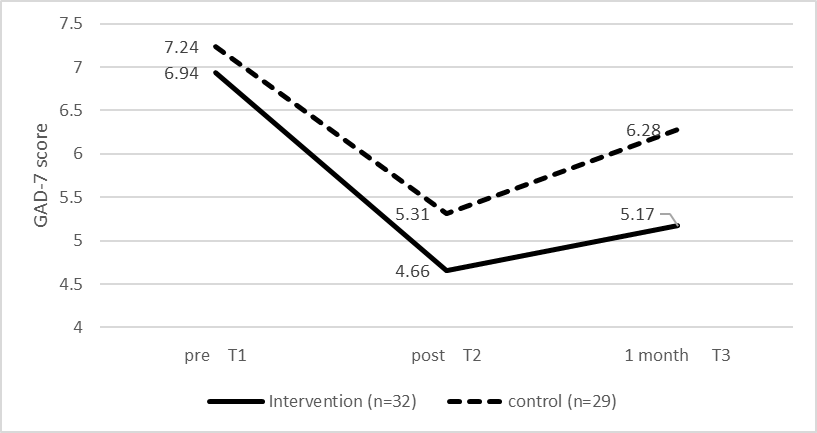
Change in the level of anxiety (GAD-7)*

Supplement: S1 Fig — (DOCX) [file pone.0281632.s001.docx]

Figure 3.

*
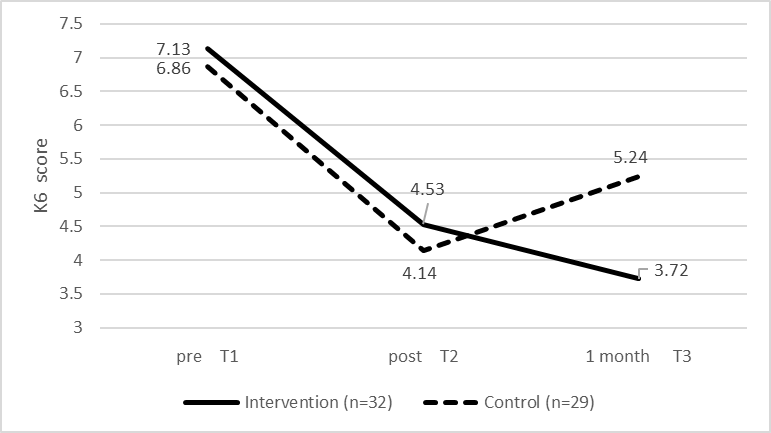
Changes in the level of depression and anxiety (K6)*

Supplement: S2 Fig — (DOCX) [file pone.0281632.s002.docx]

Figure.6

*change in the level of anxiety (GAD-7)：Multiparous*


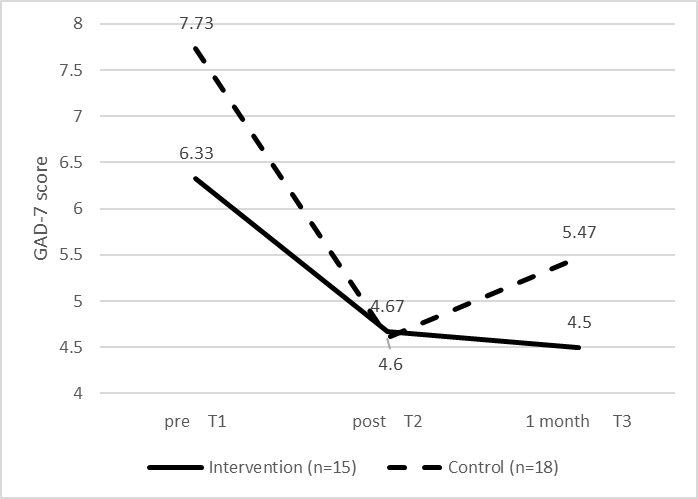

Supplement: S4 Fig — (DOCX) [file pone.0281632.s004.docx]

Figure.7


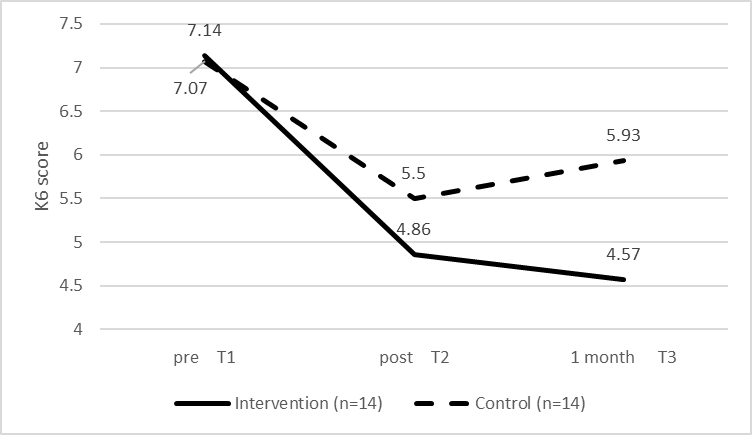
*change in the level of depression and anxiety (K6)：primipara*

Supplement: S5 Fig — (DOCX) [file pone.0281632.s005.docx]

Figure.8

*change in the level of depression and anxiety (K6): Multiparous*


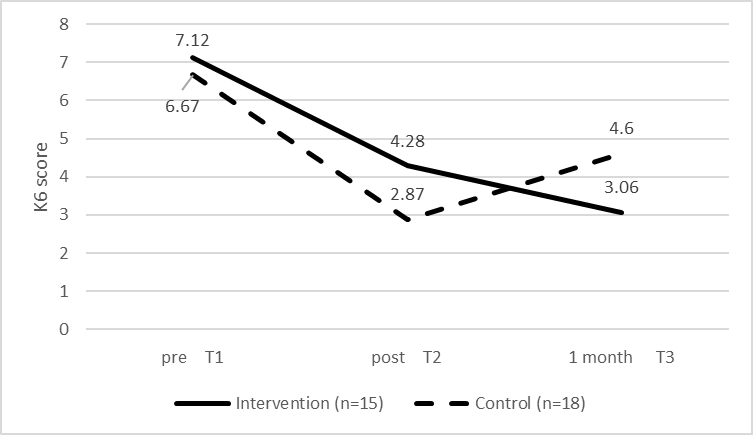

Supplement: S6 Fig — (DOCX) [file pone.0281632.s006.docx]

Figure 5.


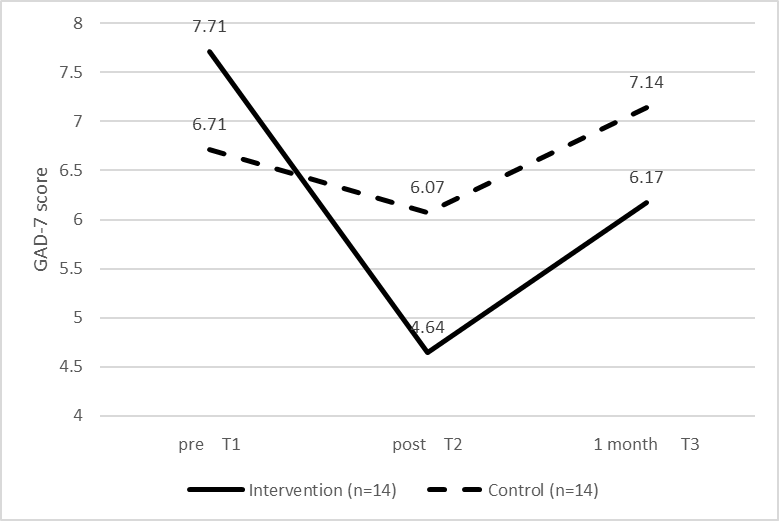
*change in the level of anxiety (GAD-7)： primipara*

Supplement: S9 Fig — (DOCX) [file pone.0281632.s009.docx]
