# Supplementary material for: Evaluation of the effect of a midwife-led online program using cognitive behavioral therapy for pregnant women at risk for anxiety disorder in Japan: A pilot randomized controlled trial
Source: PLoS One. 2023 May 10;18(5):e0281632. doi: 10.1371/journal.pone.0281632 (PMC10171600; doi:10.1371/journal.pone.0281632)
Supplement: S3 Fig — (DOCX) [file pone.0281632.s003.docx]

Figure 4.

*Change in self-efficacy for cognitive behavioral therapy response*


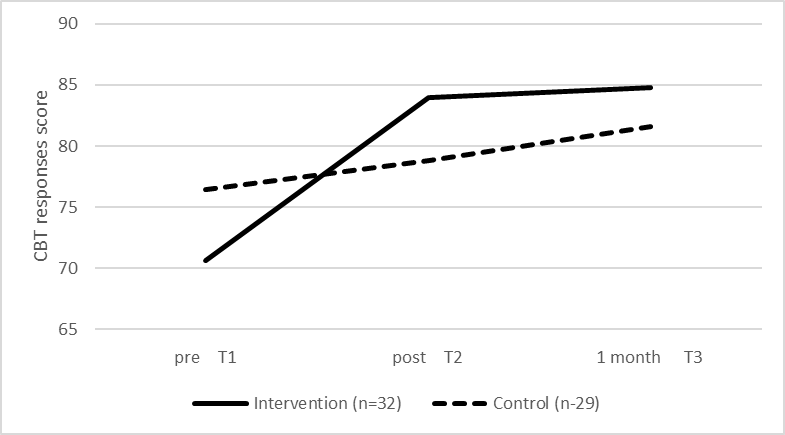
*(Self-efficacy assessment tool scores for CBT response)*
